# Macrophage: A Cell With Many Faces and Functions in Tuberculosis

**DOI:** 10.3389/fimmu.2022.747799

**Published:** 2022-05-06

**Authors:** Faraz Ahmad, Anshu Rani, Anwar Alam, Sheeba Zarin, Saurabh Pandey, Hina Singh, Seyed Ehtesham Hasnain, Nasreen Zafar Ehtesham

**Affiliations:** ^1^ Laboratory of Infection Biology and Cell Signaling, Indian Council of Medical Research (ICMR)-National Institute of Pathology, New Delhi, India; ^2^ Kusuma School of Biological Sciences, Indian Institute of Technology, Delhi (IIT-D), New Delhi, India; ^3^ Department of Biochemistry, Jamia Hamdard, New Delhi, India; ^4^ Department of Biochemical Engineering and Biotechnology, Indian Institute of Technology, Delhi (IIT-D), New Delhi, India; ^5^ Department of Life Science, School of Basic Sciences and Research, Sharda University, Greater Noida, India

**Keywords:** *Mycobacterium tuberculosis*, innate immunity, macrophage heterogeneity, phenotype switching, metabolic reprogramming, trained immunity

## Abstract

*Mycobacterium tuberculosis* (*Mtb*) is the causative agent of human tuberculosis (TB) which primarily infects the macrophages. Nearly a quarter of the world’s population is infected latently by *Mtb*. Only around 5%–10% of those infected develop active TB disease, particularly during suppressed host immune conditions or comorbidity such as HIV, hinting toward the heterogeneity of *Mtb* infection. The aerosolized *Mtb* first reaches the lungs, and the resident alveolar macrophages (AMs) are among the first cells to encounter the *Mtb* infection. Evidence suggests that early clearance of *Mtb* infection is associated with robust innate immune responses in resident macrophages. In addition to lung-resident macrophage subsets, the recruited monocytes and monocyte-derived macrophages (MDMs) have been suggested to have a protective role during *Mtb* infection. *Mtb*, by virtue of its unique cell surface lipids and secreted protein effectors, can evade killing by the innate immune cells and preferentially establish a niche within the AMs. Continuous efforts to delineate the determinants of host defense mechanisms have brought to the center stage the crucial role of macrophage phenotypical variations for functional adaptations in TB. The morphological and functional heterogeneity and plasticity of the macrophages aid in confining the dissemination of *Mtb.* However, during a suppressed or hyperactivated immune state, the *Mtb* virulence factors can affect macrophage homeostasis which may skew to favor pathogen growth, causing active TB. This mini-review is aimed at summarizing the interplay of *Mtb* pathomechanisms in the macrophages and the implications of macrophage heterogeneity and plasticity during *Mtb* infection.

## Introduction

Tuberculosis (TB), the oldest global pandemic since prehistoric times, is caused by *Mycobacterium tuberculosis* (*Mtb*) which has co-evolved with humans for around 70,000 years (1, 2). While the exact evolutionary age of *Mtb* is contentious, it has been a cause of significant concern at least since Neolithic human expansion (3–5). As per the current estimates, a quarter of the world’s population has a latent form of TB (6) and around 10% of them may develop active TB during their lifetime (7). Annually, nearly 10 million people are affected with TB which causes nearly 1.2 million deaths (8). The situation is further complicated due to increasing numbers (21%, 0.46 million) of drug-resistant TB (9). Moreover, the control and management of TB has currently been affected due to the unprecedented COVID-19 pandemic ravaging the world (10). As a result, there have been a decrease in notifications and treatment trends and an enhanced mortality for TB. The WHO has therefore flagged concerns about the retardation in the milestones envisaged for the END-TB program (11). With decreased notification and treatment trends and enhanced mortality reported, the COVID-19 pandemic has been suggested to have reversed the years of progress made in TB control efforts (11).

The reductive evolution of the genome has enabled *Mtb* to evolve into a more virulent and successful pathogen (12, 13). The reductive evolution of *Mtb* from its evolutionarily close and mildly virulent species including *M. kansasii* and *M. marinum* has been reported (14–16). The actual size of the genome in common ancestors of mycobacteria is ambiguous; hence, it remains an open question whether a large number of open reading frames (ORFs) were lost during the reductive evolution.

Macrophages are the frontline cells of innate defense and are present in every major tissue. Macrophages play crucial roles in maintaining tissue integrity, homeostasis, and wound repair and regulating inflammatory processes (17, 18). *Mtb* also makes efficient use of macrophage heterogeneity and plasticity for productive infection and dissemination. Following infection through the aerosol route, mostly alveolar macrophages (AMs) in the lungs harbor *Mtb*. To survive immune or drug pressure, *Mtb* can acquire and maintain a “metabolically slowed” latent infection phase within the macrophages, which are the primary innate immune responders for eliminating the intracellular pathogens (19–24). However, it is largely unclear which macrophage subtype(s) *Mtb* prefers for its latent residency program. *Mtb*, thriving within the macrophages, has evolved a number of mechanisms to evade or counter the host immune response (25). As a result, macrophages serve as a suitable niche for the survival of *Mtb*, making it one of the most successful pathogens (26).

Macrophages are characterized based on their functional and spatial heterogeneity. For example, Kupffer cells that populate the liver and the glial cells or microglia present in the brain are both subtypes of macrophages. Ontologically, the resident macrophages that arise from the embryonic yolk sac remain within their designated tissue spaces for a lifetime or differentiate from the bone marrow (fetal liver at the prenatal stage)-derived myeloid mononuclear cells, giving rise to mature macrophages in virtually all the tissues (27, 28).

The primary organ for TB infection is the lungs which are predominantly populated by fetal liver monocytes, bone-marrow-derived resident AMs, and self-renewing macrophages that originate from the yolk sac (27–30). In response to inflammation, the recruited blood monocytes can differentiate into AMs in the tissue microenvironment and are termed as recruited AMs (31, 32). Interestingly, in a mouse model of mixed AMs, embryonic host-derived and donor-derived postnatal macrophages displayed minor (0.1% of all the genes) yet conserved differences in transcriptomic signature and exhibited overlapping functional attributes (29). Further studies have revealed significant differences in the metabolic, proliferative, and inflammatory states of the resident and recruited AMs. Macrophages derived from the circulatory monocytes are more proliferative and pro-inflammatory, are short-lived, and derive energy primarily from glycolysis (32).

The interstitial macrophages (IMs) are relatively stable and short-lived as compared with the AMs (33) and are localized in either the alveolar interstitial or peribronchial regions (31). Recent studies have described two distinct lineages of IMs consisting of 1) Lyve1^low^MHC-II^high^ IMs with a role in antigen presentation and 2) Lyve1^high^MHC-II^low^ perivascular IMs involved in wound healing and tissue repair (31). These subsets of IMs are conserved in mice and humans (33–35).

Depending on the activation status, macrophages were initially categorized into M1 type with pro-inflammatory attributes and M2 type with anti-inflammatory features (36, 37). However, the dichotomy of M1 and M2 types is now considered an oversimplification of the complex functional heterogeneity of the macrophages (38). Recent studies have shown diversity in macrophage populations which do not exhibit typical characteristics of either the M1 or M2 sublineages (39, 40). Therefore, a dynamic classification is needed to incorporate different subsets of macrophages, which may characterize beyond the dimorphic M1/M2 paradigm. The present classification of macrophages does not account for the microenvironmental conditioning and immunological stimulus which direct M1/M2 diversification. A clear-cut demarcation of the M1/M2 subset becomes obscure due to the coexistence of diverse stimuli in the inflamed tissues (41).

**Figure 1:**
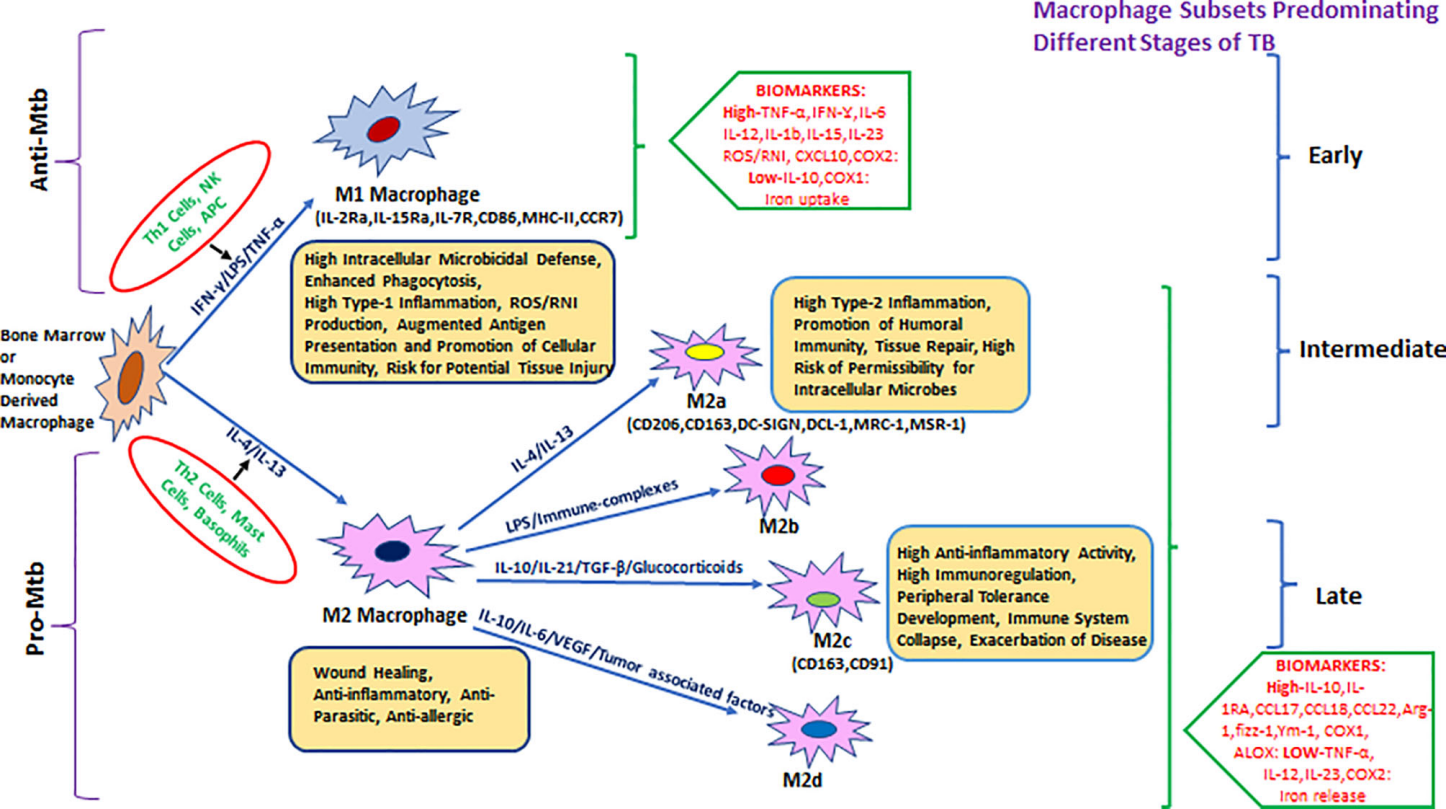
Heterogeneity of bone-marrow- or monocyte-derived macrophages and their physiological roles with reference to TB. The fate and function of recruited macrophages are generally shaped with influence from the local environment, stimulatory signals, and type of infection. Effector cells, including Th1/NK cells and APCs, displaying antigenic peptides from intracellular pathogens (or due to stimulation with LPS/IFN-γ), give rise to the M1 type of pro-inflammatory macrophages that either clear or restrain (through granulomatous response) intracellular infections including *Mycobacterium tuberculosis* (*Mtb*). M1 macrophages are generally characterized by a high level of pro-inflammatory mediators such as TNF-α, IFN-γ, IL-6, IL-12, IL-1β, IL-15, IL-23, CXCL-10, COX-2, and ROS/RNI and a low level of immune-regulatory molecules including IL-10, IL-4, TGF-β, and COX-1. In parallel, during invasion by an extracellular pathogen, Th2 cells/mast cells/basophils (or stimulation with IL-4, IL-10, IL-13, and immune complexes) act to differentiate macrophages toward the M2 state that generally ensure clearance of extracellular parasites. M2 macrophages are generally characterized by the production of a high level of IL-10, IL-1Ra, CCL17, CCL18, CCL22, Arg-1, fizz-1, Ym-1, COX-1, ALOX, etc. and a low level of TNF-α, IL-12, IL-23, and COX2 among others and further divided into various subtypes such as M2a, M2b, M2c, and the most recently described M2d. They are known for their role in promoting intracellular infections, for example, AMs in *Mtb* infection.

Hence, it was proposed to categorize macrophages based on the effector molecules they produce (**Figure 1**), for example, M(IFN-γ), M(IL-4), or M(IL-10) (42). M1-activated macrophage markers include inducible nitric oxide synthase (iNOS)/eNOS, IFN-γ, STAT-4, T-bet, SOCS3, CCR7, and CCL19/21. M1-activated macrophages have the absence (or low expression) of arginase-1/2, CD206, CD163, MerTK, STAT-3/6, Ym1/2, Fizz1, and MRC1 markers, which are predominantly expressed in M2-biased macrophages (43–48). M1 macrophages were also subcategorized as classical and innate activated macrophages, M1a and M1b, respectively (49).

M2-activated macrophages showed greater diversity as compared with the M1 macrophages. Several subtypes of M2 macrophages such as the M2a/b/c and M2d are categorized based on different activation states and associated cytokine/chemokine signaling. M2a macrophages express CD206 (mannose receptor), differentiate in response to IL-4 and IL-13 (mainly produced by Th2 cells, mast cells, and basophils), and can downregulate pro-inflammatory responses (36, 47).

M2b macrophages, activated by immune complexes and TLR agonists, produce both pro- and anti-inflammatory cytokines (49). M2b differentiation is induced by IL-1R ligand or exposure to LPS. This phenotype is marked by a low expression of IL-12 and a high expression of IL-10 which favors Th2 type immune response (50).

M2c macrophages express anti-inflammatory cytokines such as IL-10, IL-21, TGF-β, or glucocorticoids. M2c macrophages are upregulated during the scavenging activity of the cellular debris and are associated with tissue remodeling (36, 47). *Mtb-*permissive M2c macrophages that get differentiated *via* activation of IL-10/STAT-3 signaling display angiogenic characteristics and are implicated in TB pathogenesis (43, 51).

M2d macrophage phenotypes were characterized from Fra-1-mediated differentiation of RAW264.7 macrophages upon induction with tumor cells. M2d macrophages have a characteristic low expression of IL-12 and high expression of IL-6 and IL-10 and exhibit immunosuppressive features of tumor-associated macrophages (TAMs) (52). M2d macrophages are also derived by co-stimulation with TLR and adenosine receptor agonists and are characterized by high levels of IL-10, VEGF, and iNOS expression, which are independent of IL-4Rα expression (53, 54).

Despite all these findings, characterization of the exact surface markers for distinguishing monocyte/macrophage subsets is challenging due to overlapping markers and due to the existence of hybrid subpopulations co-expressing both M1/M2 markers (55, 56). Therefore, renewed attempts are required to precisely describe cell surface markers which can define and delineate different macrophage subsets.

In *Mtb* infection, the enormous macrophage heterogeneity makes it difficult to delineate the macrophage subtypes that are protective or pathogenic in nature. In *Mtb*-infected lungs, distinct macrophage subtypes such as the AMs, monocyte-derived macrophages (MDMs), and IMs have been identified (30). In the initial stage of infection, AMs act as a niche for *Mtb (* 57); later on, AMs move to the pulmonary interstitium to disseminate infection to other cell types including the recruited macrophages (58). The macrophages in murine lungs display additional heterogeneity with the presence of three distinct subsets of IMs: (IM)-1, 2, and 3 (34). Although IMs have a protective role in TB (59, 60), it is possible that some IM subsets can be potentially pathogenic which needs to be investigated. Additionally, a separate subset of lipid-rich foamy macrophages (FMs) has also been characterized in the *Mtb*-infected lungs (discussed later) (23, 61). Also, a recent study has identified distinct subsets of macrophages in the lungs of tumor-bearing mice, which do not fall under the purview of the common M1/M2 paradigm (40). The exact roles of these novel subsets of macrophage in *Mtb* infection are yet to be established and may be of significant interest in future studies.

## Sequel of Infection After Inhalation of Aerosolized *Mtb*

The AMs are the first responder cells that encounter the aerosolized *Mtb*. AMs are poor at processing and presenting antigen to the T cells and produce minimal amounts of anti-mycobacterial effector molecules including the reactive oxygen and nitrogen species (62). Depletion of AMs in mice resulted in reduced *Mtb* burden and enhanced survival of animals (63). Upon encounter with *Mtb*, AMs engulf the bacilli in a phagosome which may fuse with the acidic lysosome. This process, called phagolysosome maturation, is subverted by *Mtb* to escape lysosomal sequestration and killing (64–66). Recent reports suggest that *Mtb* has evolved to not only survive but also replicate within phagosomes (67). *Mtb* perforates the phagosomal membrane and leaks out to the cytosol of the macrophage (68–70) where it can replicate or cause necrosis of the infected cells to disseminate, thereby infecting bystander cells (71, 72). Analogous to the lytic and lysogenic phases of the virus life cycle, *Mtb* probably represents a two-stage intracellular growth model. In the first stage of intracellular growth, *Mtb* resides in the phagosome where it replicates. In the subsequent stage, it reaches the cytosol where it can replicate and disseminate. However, the mechanistic details on how *Mtb* manages to replicate in the cytosol are largely unclear and need to be explored.

*Mtb* disrupts membrane-compartment integrity through the ESX-1 (73–75) and phthiocerol dimycocerosates (PDIM) (76) dependent mechanisms, both of which are generally absent in avirulent mycobacteria including the vaccine strain *M. bovis* BCG (77). ESX-1 is a component of the *Mtb-*specific type VII secretion system (T7SS) consisting of subclusters, ESX-1–5 (75, 78). The main effector of the *Mtb* ESX-1 system, ESX-A (ESAT-6), along with its substrate ESX-B (CFP-10) can cause membrane perforation (72, 79–81). The other components of the ESX system, ESX-3, ESX-H, and ESX-G, can also block phagolysosome maturation by inhibiting the ESCRT (endosomal sorting complex required for transport) assembly (82, 83). *Mtb* can also modulate apoptotic pathways (84–86) and autophagy (64, 86–89). The ESX-1 system effectors, including ESAT-6 and espB, suppress autophagy to favor mycobacterial survival inside the host cells (90, 91). ESAT-6 is known to induce apoptosis in *Mtb*-infected macrophages by inducing ROS production (92). The role of ESAT-6 is equally established in causing membranolytic activities and necrotic death of the infected host cells (72, 79–81). AcpM (Rv2244), an acyl career protein of *Mtb*, inhibits the ROS/JNK signaling pathway to arrest macrophage apoptosis, which can have potential implications in virulence and pathogenesis of mycobacteria (93). Moreover, *Mtb*-infected macrophages attain the M2 phenotype that produces IL-10 and lowers the ER stress to block apoptosis, thereby favoring intramacrophage bacillary survival (94).

Thus, *Mtb* has evolved multiple strategies to breach the innate immune defenses and can modulate macrophages into a permissive niche for its quiescent growth. The inhibition of phagolysosome maturation, dissemination *via* translocation to the cytosol, and modulation of programmed cell death mechanisms are all part of its defense strategies.

## Macrophage Heterogeneity in TB

Samuel Behar and coworkers had demonstrated the role of innate immune cells including macrophages for the clearance of *Mtb* in an aerogenic infection model (95). The study showed a positive correlation between the augmented of protection against TB and early lymphatic dissemination of *Mtb* in resistant B6 mice, as compared with the susceptible strains. The study also showed that clearance of *Mtb* was associated with its rapid systemic spread, which causes potent immune priming and anti-mycobacterial response. Interestingly, T and B lymphocytes had no role in protection, which emphasized the crucial role of macrophages and other innate immune cells as primary defenders against TB infections (95).

Early clearance of *Mtb* has been associated with heightened innate immune responses and trained immunity (96, 97), notwithstanding the host genetic variability (98). Trained immunity, caused by epigenetic and metabolic reprogramming of innate immunity, confers cross-protection to the host against various pathogens (99). Trained immunity is non-specific and maintains a short-term memory that is independent of a somatic gene rearrangement scheme of adaptive immune cells (100). Monocytes/macrophages and NK cells are the major cells involved in trained immunity-mediated defense in TB and other infections (96, 97, 101–104).

In a study on human PBMC-derived macrophages, distinct DNA methylation patterns were observed in BCG-vaccinated responders compared with non-responders. Promoter sequences of genes responsible for immune responses showed loss of methylation, which corroborated with increased *ex-vivo* anti-mycobacterial activity (105). In mice, intravenous BCG administration led to epigenetic and metabolic reprogramming of hematopoietic stem cells, resulting in enhanced myelopoiesis at the expense of lymphopoiesis (106). Preferential myelopoiesis in BCG-immunized mice gave rise to monocytes and macrophages with “trained immunity” features that were associated with protection against *Mtb* infection *in vitro* and *in vivo (* 106 *).* In another report, priming of human monocytes (and mice) with fungal cell wall PAMP (β-glucan) resulted in enhanced protection against unrelated TB infection *via* IL-1 signaling-dependent trained immunity (107).

In mice, monocytes and macrophages display at least two distinct phenotypes based on the level of expression of Ly6c, a surface marker present on the cells of myeloid origin (108–110). Ly6c^high^ monocytes are recruited to the site of inflammation, while the Ly6c^low^ subset patrols the blood vessels for vascular integrity (110, 111). Monocytes that infiltrate the site of inflammation or injured tissues can differentiate into cells that are either pro- or anti-inflammatory, depending on the microenvironmental stimulus. Ly6c^high^ pro-inflammatory monocytes convert into anti-inflammatory M2 macrophages and affected the significant regression of the atherosclerotic plaque (112) or inflammation-induced damage of liver tissue during chronic infection caused by *Schistosoma mansoni* (113). Inflammatory Ly6c^high^ monocytes/macrophages have a protective role against *Brucella abortus* infection (114), in contrast to their detrimental role in controlling visceral leishmaniasis caused by *Leishmania donovani* (115). Although Ly6c+^(high/low)^ monocytes and monocyte-derived macrophages were significantly mobilized in TB-infected mice (111) and contributed to rBCG30 vaccine-induced protection (116), their exact role in the protection or pathogenesis of TB is yet to be fully elucidated. Infection with *Mtb* can modulate the macrophage from a pro-inflammatory to an anti-inflammatory cell type (25, 117), and the recruited monocyte-derived permissive M2 macrophages may also contribute to this pool; however, it is yet to be established. Two excellent reviews have been published on the functional and phenotypical heterogeneity of the cells of the mononuclear phagocyte system in the context of TB, which can be perused for a greater understanding of the topic (68, 118).

## Interactions of Lung Alveolar Macrophages With *Mtb*

The upper regions of the lungs are constantly exposed to particulate stimulants like dust, pollen, and organic and inorganic particles as well as microbes (29, 119, 120). Macrophages can sense the pathogen/damage-associated molecular patterns (PAMPs/DAMPs) using the cell surface pathogen recognition receptors (PRRs) and initiate appropriate immune responses for clearance or containment of an underlying stimulant. Recurrent exposure causes persistent innate immune activation in the lungs. A subset of lung macrophages plays a regulatory/suppressive role in limiting the collateral immunopathological consequences (62, 119). AMs, given their predominant immunoregulatory role, are involved in taming excessive inflammation during *Mtb* infection (30, 62, 121). Despite their host-protective roles, AMs serve as a niche for *Mtb* and help subdue immune surveillance for *Mtb* clearance (58–60, 122). *Mtb* can subvert continuous innate cell resistance by masking its crucial PAMPs beneath the stealth coat of specialized PDIM lipids on its surface (120). It was suggested that PDIMs protect the *Mtb* from recognition and killing by highly phagocytic iNOS^+^ M1 macrophages and facilitate its smooth passage to the distal regions of the lungs (120). *Mtb* traverses through the pathogen-eliminating environment in the upper respiratory tract and preferentially resides in the distal ends of the lungs, and the mechanism by which *Mtb* reaches its preferred niche is largely unclear.

A two-pronged explanation of this phenomenon has been proposed based on the zebrafish model of TB. First, *Mtb* evades scrutiny by the mycobactericidal iNOS^+^ macrophages in the lungs by using cell surface PDIM lipids in a TLR2/MyD88-dependent manner (120). Secondly, virulent mycobacteria (such as *M. marinum*) exploit a unique lipid effector, phenolic glycolipid (PGL), to secrete CCL2, a chemokine ligand for CCR2, which recruits permissive macrophages to the infection site in a STING–CCL2–CCR2-dependent manner. This same mechanism may also facilitate the transfer of *Mtb* from the lung-resident AMs to the recruited permissive monocytes/macrophages for survival and dissemination (123).

Interestingly, a transcriptional repressor coded by *Mtb* Rv3167c negatively regulates PDIM expression, and a loss-of-function mutant (*Mtb* ΔRv3167c) was demonstrated to have an enhanced ability to escape the phagosome to the cytosol with augmented autophagic and necrotic cell death (124). The observed effects were attributed to the enhanced PDIM levels and were reversed in the double deletion mutant (*Mtb* ΔRv3167c Δmmpl7) with impaired PDIM production, confirming the central role of PDIM behind the enhanced virulence (124). More recently, PDIMs have been implicated in *Mtb* infection of the epithelial/endothelial cells (125, 126). Apart from other effectors that partner with PDIM in the *Mtb* virulence program, the ESX-1 operon effectors are also determined to be essential for PDIM conferred virulence to *Mtb* (127). BCG can produce PDIM but cannot escape phagosome due to a lack of RD components including ESX-1. Exploiting this fact, it was shown that transforming BCG with ESX-1 enables it to escape the phagosome, confirming that both PDIMs and ESX-1 are required for mycobacteria to escape the phagosomes (76). Concomitantly, multiple mutant strains impaired in producing PDIMs (ΔppsD, Δmas, ΔdrrC, Δhrp1, and Δrv0712) were inefficient in secreting ESX-1 effectors, highlighting the co-dependability of PDIMs and ESX-1 system proteins for mycobacterial virulence (128).

In addition to the recruited permissive macrophages, the lungs (murine) are home to the highly heterogeneous macrophage population (34, 40). The latest insight into the heterogeneity of lung macrophages in TB has been provided in a study by Cohen et al. (58) The study revealed an unexpected role of AMs and demonstrated that AMs translocate *Mtb* away from the alveolar space to the interstitium before the arrival of recruited myeloid cells in mice lungs. This is orchestrated jointly by *Mtb* ESX-1 components and host MyD88/IL-1R/ASC-mediated inflammasome signaling. The unexpected role of *Mtb*-infected AMs in traversing the epithelial boundary to deliver bacilli to the recruited monocytes/macrophages in interstitial space has renewed the interest in redefining the macrophage realm in TB-infected lungs. Plausibly, the newly identified monocyte-derived recruited AMs (31, 32) may overlap with the population identified by Cohen et al. (58) which are responsible for *Mtb* dissemination.

## The Role of Recruited Interstitial Macrophages in TB

**Figure 2:**
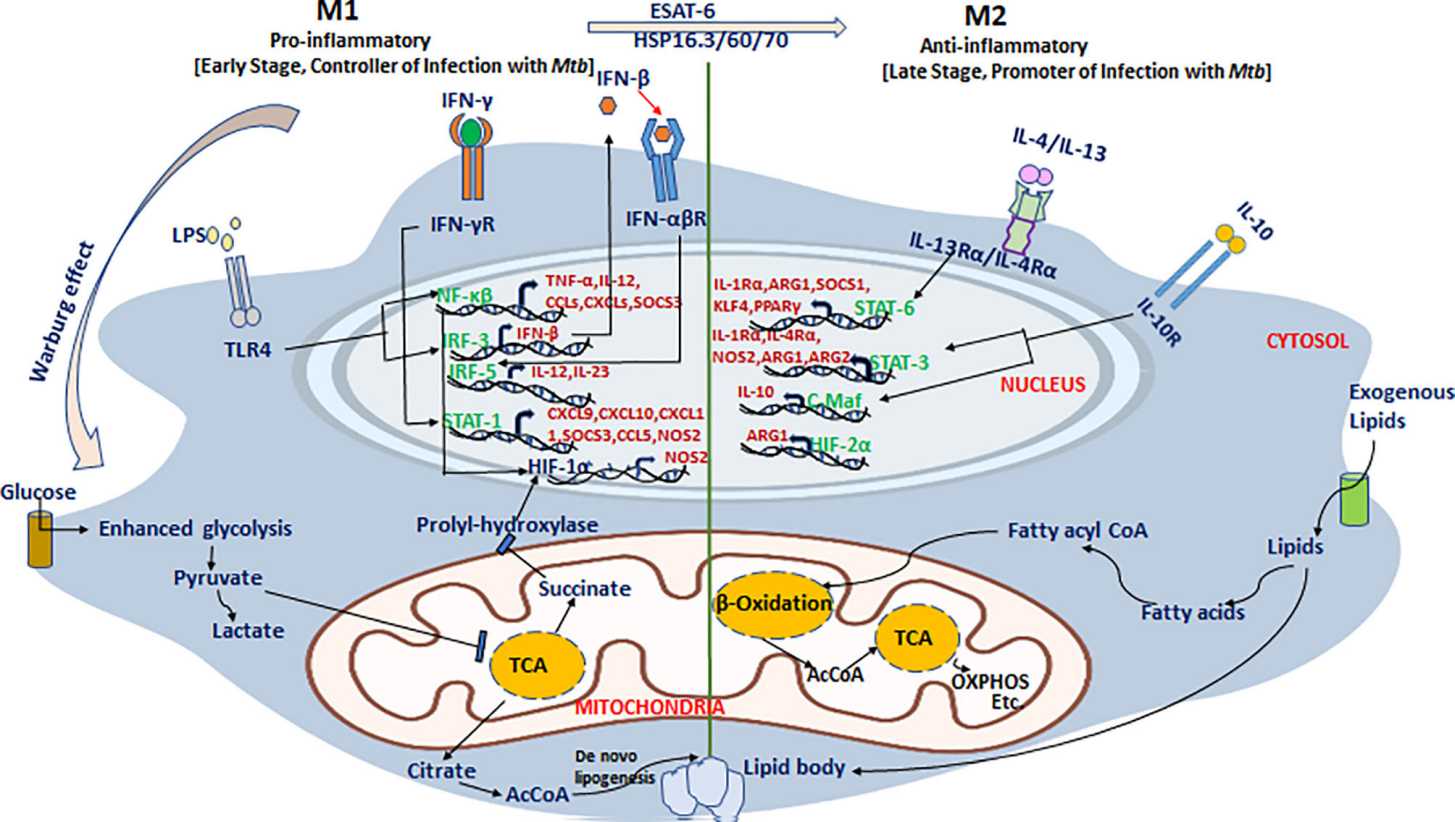
An immunometabolic circuit that dictates macrophage fate and function in tuberculosis. M1 macrophages that restrict *Mtb* proliferation are generally glycolytically active and utilize more glucose to meet increased energy demand as a result of enhanced proliferation. M1 macrophages skip the tricarboxylic acid (TCA) cycle and prefer energetically favored lactate production from pyruvate even in the absence of oxygen (Warburg effect) to which support rapid cellular turnover and generation of anti-mycobacterial oxidative burst. Instead of the TCA cycle, they utilize the pentose phosphate pathway and citric acid cycle for extra-mitochondrial utilization of available fatty acids to meet increased energy demands to support activation of pro-inflammatory genes including NF-κβ, IRF-3/5, STAT-1, and HIF-1α and their downstream effectors such as TNF-α, IL-12, IFN-β/γ, CCL-5, and CXCL-4/9/10/11 to restrict *Mtb* growth. In stark contrast, both AMs and M2 macrophages that support *Mtb* persistence/proliferation elect cost-inefficient mitochondrial oxidative phosphorylation (OXPHOS) and fatty acid oxidation (FAO) pathways to meet cellular demands. *Mtb* exploit these pathways to hijack host cells and utilizes the host’s own lipids to thrive in hibernation for longer periods. In M2 macrophages, it induces the cellular expression of anti-inflammatory mediators including STAT-3/6, C-Maf, and HIF-2α that stimulate the production of downstream anti-inflammatory effectors including IL-10, IL-1Rα, IL-4Rα, Arg-1/2, and PPAR-γ among others and thus make a permissive environment for *Mtb* growth as well persistence.

Recently, Russell and coworkers defined the dynamics, phenotype, and role of different macrophage subsets in the lungs of *Mtb*-infected mice using fluorescent *Mtb* reporter strains and macrophage transcriptomics data (59, 60). They demonstrated that *Mtb* predominantly inhabits the lung’s resident AMs for their unchecked growth, while monocyte-derived macrophage subsets (IMs) restrict *Mtb* survival in the lungs. It is apparent that *Mtb* faces less stress in AMs than in the IMs, making AMs a permissive niche for bacilli. These two macrophage subsets have shown distinct inflammatory states as a result of adopting different metabolic programs. The immunometabolic circuit of TB-loaded macrophages is described in **Figure 2**. It was demonstrated that AMs predominantly sustained fatty acid oxidation (FAO) and oxidative phosphorylation (OXPHOS), while pro-inflammatory IMs were committed to glycolysis for their sustenance. It has been shown that recruitment of macrophages is CCL2 dependent, and in CCL2-deficient mice, the migration and transformation of circulatory monocytes into the lung’s IMs is abolished with concomitant loss of *Mtb infection control (* 59 *).* The results were divergent from the earlier reports in the zebrafish infection model of *M. marinum*, where the *Mtb* lipid PGL exploits the host CCL2–CCR2 axis to recruit permissive macrophages which serve as a niche for *Mtb* growth (120, 123). These divergent findings need to be interpreted with caution as both studies were done in different model systems with different species of virulent mycobacteria (59, 120). This example also insinuates caution while translating findings from one model system to another and in humans ultimately. Additionally, it should also be noted that majority of the studies were carried out on cell lines *in vitro* which are not the exact representation of the complex macrophage landscape in tissues (129).

Nonetheless, the exact deciding factor(s) for the fate of mycobacteria-loaded macrophage is unknown. It could be dependent on the ontogeny of macrophages (embryonic or bone-marrow-derived) or the tissue microenvironment. The bone-marrow-egressed monocyte-derived macrophages are important innate responder cells during *Mtb* infection. The monocyte mobilization to *Mtb*-infected lungs is the result of emergency monopoiesis in bone marrow, rather than recruitment from blood (111). The classical Ly6c^high^ monocytes can replenish the local macrophage reservoir in *Mtb*-infected lungs, differentiate into protective IMs, and support local innate resistance against *Mtb (* 59 *).* Recruited inflammatory monocytes (Ly6c^high^) enter the lung parenchyma and give rise to multiple subsets of macrophages and DCs. Among them, CD11c^high^CD11b^+^Ly6c^high^ DCs are involved in the transport of *Mtb* to the lungs draining mediastinal lymph nodes (111). Interestingly, *Mtb* hampers antigen processing and presentation by delaying the migration of local DCs to the draining lymph nodes during early infection. It uses this strategy as a means to delay the arrival of adaptive immune cells in infected lungs by ~10–12 days in mice (130) and up to 6 weeks in humans (7) to establish a chronic infection. Plausibly, the recruited monocyte-derived DCs in *Mtb*-infected lungs may represent a host strategy to pose a counter to *Mtb*-mediated suppression of adaptive immune priming of naive DCs and T cells in the local lymph nodes.

## *Mtb* Promotes Biased M2 Shift in Macrophages to Dampen Host Immune Defense

Usually, bacterial pathogens induce a pro-inflammatory immune milieu that modulates macrophage polarization toward the M1 state, which can clear acute infections (47). Intracellular bacterial pathogens including *Mtb* (61), *Listeria monocytogenes* (131), and *Brucella abortus (* 114) modulate macrophages toward an anti-inflammatory M2 state. This M1/M2 bias synchronizes intracellular bacterial fitness to favor either persistence or proliferation. Macrophages may kill *Mtb* or serve as a reservoir for intracellular *Mtb* persistence and proliferation and may be involved in immune regulation as well (87). M1–M2 switch in macrophages skews the transition from acute to chronic infection. *Mtb* has evolved strategies to escape M1-activated macrophage killings and drive phenotypic switch to the M2 polarization state to promote chronic infection (43, 117).

In an acute *Mtb* infection, the macrophage switches to the M1-polarized state and activates downstream PRR signaling to produce multiple host-protective effectors including reactive oxygen (ROS) and nitrogen (NO) species (47) and host defensins such as cathelicidin-related anti-mycobacterial peptide (Cramp) (132), all of which help kill *Mtb*. M1 macrophages initiate granuloma formation as a means to contain the infection. Overt M1 activation induces an exaggerated pro-inflammatory response that hampers tissue homeostasis and granuloma intactness. As a result, M1 macrophages skew toward M2 polarization to regulate inflammation and to promote tissue repair during *Mtb* infection progression in the chronic phase (121, 133). This plastic behavior during macrophage polarization depends on the distinct immune microenvironment, a result of extensive metabolic reshuffling. Macrophages undergo a metabolic shift from aerobic glycolysis to mitochondrial OXPHOS and glutamine metabolism (134) cater to its energy requirements during *Mtb* infection. In the lung lesions of TB patients, M1 macrophages are mostly present in non-granulomatous sites, whereas M2 macrophages predominate necrotic as well as non-necrotic granulomatous zones, which point to the principal role of M2 macrophages in granulomatous reactions as well as possible M1–M2 transition during infection (135).

ESAT-6 plays a significant role to skew M2 polarization for *Mtb* survival during an infection (94). Serine proteases (thrombin and trypsin) (136) and heat shock proteins Hsp70 (DnaK), Hsp60, and Hsp16.3 of *Mtb* are also involved in M2 polarization to shield the bacteria from the host immune pressure (137–139). ER stress-mediated apoptosis is typical in M1 macrophages to control infection (94). Apoptosis induces a drastic T-cell response which is less favorable for *Mtb* growth and dissemination (140). In contrast, cell death *via* necrosis, together with M2 shift in early infected macrophages, provides a permissive environment for *Mtb* proliferation (47). Thus, macrophage polarization is a crucial mechanism of virulence and pathogenesis of *Mtb* with contribution of multiple host and pathogen effectors, which needs to be explored in greater detail.

## Differential Metabolism in M1/M2-Polarized Macrophages

Macrophage metabolic plasticity plays a significant role in disease pathology (141). Macrophage polarization defines the distinct metabolic profile of macrophages that drives differential macrophage activation (142). M1 cells actively use aerobic glycolysis (known as the Warburg effect in cancer cells) for bioenergetics and biosynthetic intermediates and also induce pentose phosphate pathway for ROS production. In contrast to M1, M2 cells prefer mitochondrial OXPHOS and glutamine metabolism as carbon and nitrogen sources, similar to the resting macrophages (36, 134, 143, 144). Arginine metabolism is involved in the differential regulation of macrophage polarization. L-arginine is a common substrate for iNOS as well as arginase-1. M1 macrophages produce iNOS that catabolizes arginine into L-citrulline and NO, which mediate cytotoxicity to control bacterial infection. Arginase-1 produces polyamine, L-ornithine, and urea linked to the wound-healing activity of M2 macrophages (145, 146). During TB, both iNOS (M1) and arginase (M2) compete for arginine, and this competition eventually shapes the dominant macrophage phenotype and functionality (M1 or M2), which is crucial for mycobacterial control (134). Lipid metabolism is also found to be differentially regulated in macrophage polarization. The COX-2 gene is upregulated in M1 macrophages, whereas COX-1 is upregulated in M2 macrophages (147). Membrane phospholipid-derived downstream metabolites of arachidonic acid (AA), prostaglandins, leukotrienes, and lipoxins play a diverse and essential role in shaping the immune response in TB, primarily by modulating cell death type (148–152). The delicate balance between the production and bioavailability of lipoxin A-4 (LXA-4) and prostaglandin E-2 (PGE-2) largely dictates the programmed death in *Mtb*-infected macrophage (148, 149). *Mtb* causes macrophage necrosis by inducing LXA-4 production and inhibiting PGE-2 (148). PGE-2 production is critical for apoptotic death of macrophage (148) and regulates anti-TB immune response primarily through its engagement with E prostanoid-2 (EP-2) receptor (153). While EP-2 promotes type 2 immune response (154), mice deficient in EP-2 develop pathogenic Th17 and Treg responses associated with poor clearance of *Mtb*.

Iron utilization is a classic hallmark of *Mtb* infection with the predominant role of PE/PPE proteins (155–161). M1 macrophages can sequester iron by high ferritin and low ferroportin ratio and also via heme uptake to maintain the bacteriostatic effect. This scenario is reversed in M2 macrophages, which reduces iron storage and releases iron to favor tissue repair and cell remodeling (162, 163). Heme is a cellular reserve of iron and *Mtb* is known to utilize heme for intracellular subsistence (159, 161, 164, 165). Heme oxygenase-1 (HO-1) has been reported to be upregulated in *Mtb-*infected mice, rabbits, and macaques (166). The induction of HO-1 is dependent on NADPH oxidase-dependent ROS production and nuclear translocation of the transcription factor NRF-2, which is mediated by ESAT-6 (166). Inhibition of HO-1 controls *Mtb* infection in macrophages *in vitro* and in mice *via* upregulation of NOS-2/IFN-γ and enhanced expansion of T lymphocytes (167, 168), which might be the result of macrophage reprogramming toward the pro-inflammatory M1 state. Furthermore, the essential *Mtb* gene *ripA* rewires the macrophage metabolism toward glycolysis by inhibiting mitochondrial oxidative phosphorylation (169). The glycolysis induced in RipA-treated RAW264.7 macrophages indicates RipA-mediated modulation of the macrophage phenotype which, however, was associated with poor intramacrophage growth control of recombinant *Mycobacterium smegmatis* expressing *Mtb* RipA. This observation, while underlining an important role of *Mtb* RipA, also motivates fresh investigations into the macrophage modulatory roles of unexplored *Mtb* effectors beyond common ESX system proteins.

## Macrophage M1/M2 Paradigm: Conflicting Evidence From Human TB

Much that has been said and known about the heterogeneity of macrophages in TB is learned from animal models, primarily mice. Very few studies are available or being done that aim to characterize monocyte/macrophage dynamics in human lungs, a primary site of *Mtb* infection. This might be, in part, due to the ethical and anatomical challenges associated with collecting tissue/BAL samples from human lungs. While the M1/M2 dichotomy is largely established in the lungs and spleen of mice, the macrophage identity is not as demarcated in humans, and considerably divergent phenotypical heterogeneity has been reported in macrophages from various anatomical origins (55, 56, 122).

Emerging reports have indicated the presence of mixed macrophage phenotype (co-expressing both M1/M2 markers) in a range of conditions (55, 56, 170). AMs from human lungs, obtained from two geographically distinct populations of the UK and Malawi, were immunophenotyped. These AMs displayed mixed expression of M1 (CD80/86) and M2 (CD163/206) markers, which challenges the established dichotomy of the macrophage phenotypical identity (56). Another study has reported circulatory macrophage (CD204^+^CD163^+^CD206^+^TLR4^+^CD80^+^CD86^+^) and monocyte (CD14*^+^* CD206^+^CD163^+^CD204^+^TLR4^+^CD80^+^CD86^+^) populations that expressed both M1/M2 markers in systemic sclerosis patients and in interstitial lung disease (ILD) (55). In the context of TB, Lavelette and colleagues used microarray and qRT-PCR-based sequential approach to demonstrate differential immune response trajectories in BAL macrophages, obtained from human TB patients infected with clinical *Mtb* strains (170). Interestingly, differential response to two clinical *Mtb* strains belonging to LAM (Latin American and Mediterranean) lineage in pulmonary (the lung’s AMs) and extrapulmonary (splenic macrophages or SMs) sites was observed, with AMs showing a predominantly pro-inflammatory phenotype and SMs displaying a largely attenuated response. Moreover, when compared with uninfected macrophages from healthy controls, the TB-infected AMs displayed an attenuated transcriptomic response and regulation of critical gene sets related to anti-TB responses including ISGs, IFITs, and GBPs (IFN pathway), AIM2 (inflammasome) FCGR1A (fc receptor pathway), and TREM (myeloid receptor pathway), which suggest modulation of macrophage-specific immune responses. In addition to the tissue-specific macrophage responses, the two clinical isolates (UT127 and UT205) induced contrasting macrophage transcriptomic responses in human macrophages (22 and 5 genes induced, respectively). Differential responses were attributed to the altered virulence profile of the two isolates, despite belonging to the same lineage (LAM) (171), which suggest altered host immune trajectory as a plausible function of intrahost microevolution of the *Mtb* bacilli. However, the drug resistance profile of these isolates (which is unknown) might have provided additional correlates to the observed differential responses. Nonetheless, of the two isolates, one induced apoptotic cell death (UT127), while the other triggered necrosis (UT205), which itself attests to their altered virulence profile. Interestingly, the one that induced necrosis (UT205) displayed attenuated transcriptomic responses in macrophages as well, suggesting its higher virulence and immunosuppressive abilities.

In a latest attempt to characterize the spatial heterogeneity of macrophage identity and function at the primary site of TB infection, Pisu et al. recently defined at least four different subsets, each of the interstitial macrophages (IM 1–4) and alveolar macrophages (AM 1–4), in human bronchoalveolar lavage (BAL) and in mice lungs (122). The study, employing single-cell transcriptomic analysis of macrophages infected with fluorescence reporter strains for intramacrophage *Mtb* fitness, clearly defined substantial plasticity among lung macrophages. Both *Mtb-*permissive and *Mtb-*restrictive macrophage subsets were spotted among the lung’s resident AMs (AM2 being the most restrictive) and recruited IMs (IM2 being the most permissive). In addition, the study also identified CD11c^low^ IMs/AMs in the lungs as predominantly permissive to *Mtb* growth which allows the development of drug tolerance as well (122). Of note, IMs and AMs were labeled restrictive and permissive, respectively, to *Mtb* growth in a previous study by the same group (60). Nevertheless, these lines of evidence provide important insights into the macrophage heterogeneity and plasticity in TB-infected lungs from mice and humans. These data clearly suggest that both the IM and AM sublineages of *Mtb*-infected macrophages contain permissive and controller subpopulations. It is therefore time to reconstruct the simplified dimorphic view of macrophage heterogeneity and to assess the enormous phenotypical and functional polymorphism present in *Mtb*-infected macrophage which may have far-ranging implications.

In addition to macrophage ontogeny, significant heterogeneity in *Mtb*-infected macrophages has been suggested to be due to the genetic differences among the infecting strains and lineage (171–174). *Mtb* isolates belonging to the hypervirulent modern strains have been associated with a lower inflammatory response *in vitro* by human or murine macrophages due to the predominance of anti-inflammatory M2-like monocytes/macrophages (175, 176).

## Macrophage M2 Polarization and Drug Resistance in *Mtb*

Classically activated M1 polarization displays pro-inflammatory and antimicrobicidal activities (129). In contrast, alternatively activated M2 polarization plays a pivotal role in circumventing host immune defense during *Mtb* infection and may result in MDR/XDR-TB treatment failure to favor persistent infection (135). M1/M2 polarization of macrophages has a crucial role in the progression or regression of TB infection as a result of pro- or anti-inflammatory responses they exert, respectively. It was recently demonstrated that the M2 polarization rate and the M2 to M1 polarization ratio were significantly higher in the MDR/XDR-TB group as compared with the drug-susceptible TB group (177), reflecting the crucial role of macrophage polarization in drug resistance development. Thus, it may be an attractive host-directed avenue to modulate macrophage phenotype in drug-resistant (or even drug-sensitive) TB infection, with or without chemotherapy.

## Macrophage Plasticity in *Mtb* Infection

The majority of macrophages are not terminally differentiated and are poised to reprogram either homeostatically or in response to infection and other stimuli such as cytokines, growth factors, hormones, small molecules, and metabolites including prostaglandins and leukotrienes. In TB-infected mice, recruited macrophages have a dynamic expansion and differentiation program, even during the chronic stage of infection (111). Moreover, substantial diversity and plasticity of macrophages have been observed in tuberculous granuloma (46, 178) which is spatially organized (133). The imbalance of this spatially organized granulomatous structure, required for containing *Mtb*, is reminiscent of progression to active TB and is largely dictated by macrophage polarization metrics in the granuloma microenvironment (179). *Mtb* transforms macrophages into multiple subtypes through a variety of mechanisms and exploits them for its survival.

## Macrophage Reprogramming Into Epithelioid Cell, Multinucleated Giant Cell, and Foamy Cell

The macrophage at the core of granuloma undergoes a series of morphological changes including epithelioid cell differentiation to form “epithelioid” cells like macrophages. These epithelioid cells can further develop into multinucleated giant cells (MGCs), probably due to cell–cell fusion or cytokinesis failure (180–182). One of the well-characterized virulence schemes of *Mtb* is the modulation and differentiation of macrophages into lipid-rich foam cells (23, 51, 61, 183) that serve as a niche for *Mtb* persistence. In *Mtb* infection progression, macrophages convert into foam cells by importing and accumulating host lipids, mainly low-density lipoproteins (LDLs) and cholesterol (23, 51, 61, 183–185). In the following subsections, we will be discussing the origin and functionality of these macrophage subtypes which have been extensively studied and characterized for their role in TB progression.

### Epithelioid Cells or Histiocytes

*Mtb* reprograms macrophages for granuloma formation *via* E-cadherin-dependent mesenchymal–epithelial transition. Epithelioid cells are characterized as hypertrophic, flattened in appearance, containing diffused cytoplasm and elongated nuclei with the interdigitating cell membrane, which enable the cell to stick and form an epithelioid barrier to persistently capture available antigen (186). These inflammation- or infection-induced histopathological transformations in macrophage microanatomy resemble an epithelial cell, thus acquiring the name epithelioid cells or histiocytes (187). Macrophage-derived epithelioid cells form the central scaffold of organized granulomas meant to be less accessible to immune cells and, thus, provide a favorable niche for mycobacterial persistence (186, 188–190). E-cadherin expression in motile mesenchymal cells induces transformation into the epithelial structure *via* the process of mesenchymal–epithelial transition (or *vice versa*) in tissue development (191) and cancer (192). Cronan et al. demonstrated that epithelial reprogramming is analogous to the mesenchymal–epithelial transition and is conserved within the tuberculous granuloma of mice and humans (186). They confirmed that the macrophage forms adherence junctions, desmosomes, and tight junctions as a prestructure for stable granuloma formation and speeds up infection trajectory. Host angiogenic signaling has also been implicated in macrophage epithelioid transition and formation of tuberculous granulomas with an important role of vascular endothelial growth factor-receptor (VEGF-R) (193). Therapeutic inhibition of granuloma vascularization and angiogenesis using VEGF-R antagonists not only reduces *M. marinum* infection in zebrafish but also synergizes with the anti-TB drugs rifampicin and metronidazole to improve bacillary clearance (193). Recently, signal transducer and activator of transcription (STAT)-6 signaling is found to be absolutely necessary for macrophage epithelialization and granuloma formation (194). By analyzing single-cell RNA-sequencing data from *M. marinum* and *Mtb* granulomas from zebrafish and macaques, respectively, the study concluded that the strong type-2 signaling mediated *via* the IL-4R/STAT-6 derives macrophage epithelialization and granuloma formation and is largely unaffected by the presence of robust type-1 immune signals.

### Multinucleated Giant Cells

MGCs are polykaryons of monocytic origin, where multiple monocytes (or macrophages) fuse and differentiate into specialized MGCs (180, 195). Macrophage fusion occurs in the granulomatous region to form MGCs during mycobacterial infection. *Mtb* cell wall lipids, trehalose dimycolate (TDM), and lipomannan (LM) have been described to derive the formation of MGCs (183). In addition, macrophage exposure to cytokines IL-4 and IL-13 induces MGC formation (180). MGCs also possess a specialized ability to uptake large and opsonized complement particles mediated by CR3 signaling (196). However, in the context of mycobacterial infection, it was reported that MGCs contain very few bacilli and may be unable to phagocytose invading bacilli (190, 197). Classical and alternatively activated MGCs have been reported in acute and chronic TB infections, respectively (195). It is thus possible that MGCs typical of M2-type macrophages may enable rapid progression of chronic infection in TB. However, due to the paucity of credible evidence regarding the defined role and function of MGCs, they may continue to be considered a pathological hallmark of mycobacterial and other granulomatous infections.

Interestingly, the differentiation of MGCs has been shown to occur only in virulent *Mtb* complex organisms and not with other avirulent mycobacteria (197). MGCs are integral to granuloma formation and have a role in maintaining latency or conferring tolerance to *Mtb.* Recently, MGCs have been suggested to have originated from the common monocyte progenitor (CMoP) or inducible monocyte progenitor (iMoP) population in circulation (198). Their poor phagocytic activity and absence in disseminated TB during immunosuppression is intriguing and suggestive of their role as a niche for latent *Mtb (* 198 *).* MGCs contain plenty of cholesterol and other fatty acids, a preferred energy source for intracellular *Mtb* persistence *(* 184 *).* Moreover, TLR2-mediated *Mtb* modulation of macrophages induces excess NO production, leading to DNA damage and impaired p53 function and consequential establishment and differentiation of permissive MGCs (199). While these reports highlight that MGCs serve as a niche for mycobacterial survival, some earlier reports have demonstrated the protective role of MGCs in TB. Specifically, cytokines IFN-γ and IL-3, in combination with some other factors, have induced MGCs that limit mycobacterial infection *in vitro* (200, 201). In the face of these contrasting reports, more studies are required to assess the exact role of MGCs in mycobacterial pathogenesis, particularly in the context of humans. This gap in knowledge about the functional role of MGCs is an active area for future research.

### Foamy Macrophages

FMs are lipid-enriched macrophages formed due to uptake of LDL or oxidized LDL *via* LDL receptor or scavenger receptor-A (SR-A) and CD36, respectively, in response to TLR2 activation by mycobacterial components, pro-inflammatory chemokines, and cytokines (23, 61, 183, 202). The lipid-rich environment of FMs allows *Mtb* transition into the latent phase with ample access to nutrients in the form of intracellular lipids (203).

FMs can also arise through phagocytosis of platelets by monocytes. Platelets, when co-cultured with monocytes in the presence of mycobacteria, induce the formation of multinucleated giant foam cells (185). Interestingly, despite their increased phagocytic activity and BCG uptake, FMs display predominantly the M2 phenotype and produce IL-10 abundantly (185).

LDL and lipid-loaded platelets break down into triacylglycerol, phospholipids, and cholesterol (183). In mycobacterial infection, cholesterol gets accumulated within the macrophages in the form of lipid droplets or effluxes *via* ATP-binding cassette (ABC) transporters (183, 204). The ABC transporters, ABCA1 and G1, are key mediators of cholesterol efflux and their absence exacerbates FM formation (183). Accumulated cholesterol also modulates inflammatory responses by producing leukotrienes and prostaglandins (183, 205, 206). *Mtb* also utilizes the host cholesterol using the *mce4* locus (analogous to mammalian ABC transporters) for its survival and persistence during the chronic phase of infection, which supports its preference to induce differentiation of host lipid-rich macrophages (184).

In addition, the triacylglycerol synthase 1 (Tgs1) of *Mtb* helps in the accumulation of triacylglycerol (TAG)-derived fatty acids/triglycerides in macrophages (207). *Mtb*’s cell wall long-chain fatty acid and oxygenated mycolic acid induce human macrophage differentiation into FMs, which serve as a nutrient reservoir for the persistence of dormant *Mtb* for longer periods (22).

## The Heterogeneity and Plasticity of Macrophages in Tuberculous Granuloma

Small aerosol droplets containing *Mtb* from infected carriers enter the lungs through inhalation where it is phagocytosed by AMs (26). *Mtb* remains unhindered within AMs and recruits permissive macrophages through coordinated usage of surface-expressed lipid PGL/PDIM (120). PDIM is a cell wall-derived component present exclusively in the pathogenic strains of mycobacteria (208, 209) and all clinical isolates of *Mtb (* 26 *).* The conical shape of PDIM augments membrane fusion to allow efficient *Mtb* uptake *via* endophagocytosis (210). PDIM is transferred from the *Mtb* cell wall to the macrophage’s lumen through the formation of PDIM aggregates in transient membrane stalks and enhances the non-bilayer phase followed by an endocytic uptake and phagosome formation (210). The phagosomal encasement consists of both the host and *Mtb* lipids, where *Mtb* survives for an extended period and disseminates to distant locations (210, 211). *Mtb*, once able to invade AMs in the upper airways, finally settles down to the extreme ends of the lungs (26, 120). Here, it replicates and triggers necrotic death of the infected cells, thereby infecting bystander cells (30, 71) and causing a cascade of immune reactions (212). These local inflammatory reactions and release of myriad chemokines/cytokines recruit neutrophils (30), MDMs (111), and DCs (213) that engulf and transport *Mtb* to the draining lymph nodes to activate T-cell-mediated adaptive immune response (130). By the time the adaptive immune cells get activated and reach the infected lungs, the cells of the innate immune system and local non-immune cells cordon the *Mtb* in a granulomatous structure, where adaptive T and B cells further add to make the outermost layer (190). Thus, granuloma forms during the critical time window when innate immunity fails to contain the growing *Mtb* burden, following initial exposure, and adaptive immunity cannot respond within time. Traditionally, granuloma has been seen as a host-protective measure to contain *Mtb* infection (133, 214); however, growing evidence also suggests its pro-mycobacterial role (186, 188–190, 194).

Macrophages, especially AMs, act as a perfect ecosystem for intracellular adaptation of *Mtb* and to achieve persistent infection. *Mtb*-encapsulated granulomas are a highly organized structure containing a pro-inflammatory core and surrounding periphery with a strong anti-inflammatory signature (133, 214), thus holding a pro- and anti-inflammatory balance to keep itself intact and non-disseminative. The formation of caseous necrotic granuloma is the hallmark of uncontrolled *Mtb* infection and TB disease progression (190, 215). The classic necrotic granuloma has a necrotic core containing extracellular bacteria surrounded by epithelioid histiocytes and macrophages and an outer cuff of macrophages intermixed with lymphocytes (214). As granuloma matures, a number of cell-like granulocytes, monocytes, DCs, B cells, T cells, NK cells, and fibroblasts migrate to the structure and surround the macrophage core (141, 216, 217). This may cause exaggerated and uncontrolled inflammation which induces pathophysiological changes at the final stage of granuloma formation and is associated with tissue damage and morbidity (141). In a C3HeB/FeJ mouse model of tuberculous granuloma, the central caseous necrotic regions are reminiscent of immune defense and restrain the TB bacilli. However, dysregulated inflammation-induced neutrophil and lymphocyte infiltration causes liquefaction of packed granulomas that led to mycobacterial dissemination and development of active TB (218, 219).

Cytokine signaling mediates the conversion of macrophages into heterogeneous phenotypes within the granuloma encasing TB bacilli. A computational model defines the metrics of macrophage polarization as a function of cytokine signaling (179). It examined the ratio of the temporal expression of STAT-1 and NF-κβ (pro-inflammatory) to STAT-3 (anti-inflammatory) in the macrophage and concluded that the expression level of NF-κβ can dictate macrophage or granuloma polarization and outcome in TB, whether protective or disseminative.

In an *in-vitro* differentiated model of tuberculous granuloma from human MDMs, M1 polarization of macrophages predominated the early stage of granuloma formation and decreased over time, while M2 polarization gradually increased during the late stage of granuloma formation (135). The study also utilized lung tissues from TB patients to show predominant M2 polarization of macrophages in necrotic and non-necrotic granulomatous lesions, whereas non-granulomatous sections were mixed, populated with both M1 and M2 macrophages (135). Thus, tuberculous granulomas are highly plastic with spatial and temporal heterogeneity which dictates the eventual outcome of *Mtb* infection.

Although the plasticity of macrophages has long been appreciated, this dogma is getting challenged based on recent epigenetics and single-cell transcriptomics studies that hypothesize that the plasticity of macrophages is lost due to an extended residency in a particular tissue type or restricted to favor tissue homeostasis (31) or reprogram epigenetically depending on the tissue microenvironment and local inflammatory and metabolic signals during steady state or inflammation (220, 221).

## Concluding Remarks

The co-evolution of *Mtb* and its human host is in progress from prehistoric times (2). Despite numerous efforts, *Mtb* eradication is still a far-reaching target due to multiple factors. As macrophages are considered to be the primary niche for replication and persistence of *Mtb*, macrophage heterogeneity and plasticity allow them to be in multiple phenotypic and metabolic states which are skewed in favor of *Mtb* in the granuloma. However, recent reports emanating from epigenetics and single-cell transcriptomics studies have contradicted the established dogma about the plasticity of macrophages. Fresh evidence suggests that the plasticity of macrophages is lost due to an extended residency in a particular tissue type or is restricted to favor tissue homeostasis (31). Studies aimed at defining the actual dynamics of complex transitional states of macrophage populations based on tissue microenvironment and epigenetic landscape will be key to pinpoint the macrophage subsets as protective or pathogenic in TB. Nonetheless, strategic manipulation of macrophage activity and its phenotypic states can be effective to counter *Mtb.* Also, this may open new frontiers to clear off the persisting reservoir of *Mtb*, which is a source of continued supply of new infection cycles.

## Author Contributions

FA, NE and SH conceived the idea of this review. FA, AR, AA, SZ, SP, HS, SH and NE have contributed to the writing of this manuscript. All the authors have reviewed and approved the final manuscript. SH and NE are co-corresponding authors.

## Funding

SH and NE are supported by the North-East Grants BT/PR23099/NER/95/632/2017 and BT/PR23155/NER/95/634/2017 of the Department of Biotechnology, MoS&T, GoI. Intramural funds of the ICMR-National Institute of Pathology are gratefully acknowledged.

## Conflict of Interest

The authors declare that the research was conducted in the absence of any commercial or financial relationships that could be construed as a potential conflict of interest.

## Publisher’s Note

All claims expressed in this article are solely those of the authors and do not necessarily represent those of their affiliated organizations, or those of the publisher, the editors and the reviewers. Any product that may be evaluated in this article, or claim that may be made by its manufacturer, is not guaranteed or endorsed by the publisher.
